# Supplementary material for: Predicting microbial community compositions in wastewater treatment plants using artificial neural networks
Source: Microbiome. 2023 Apr 28;11:93. doi: 10.1186/s40168-023-01519-9 (PMC10142226; doi:10.1186/s40168-023-01519-9)
Supplement: Supplementary file 2 — Additional file 1. Supplementary analysis. Grouping and Comparisonof ASVs>10%. [file 40168_2023_1519_MOESM1_ESM.docx]

Additional file 1 of the article:
**Predicting microbial community compositions in wastewater treatment plants using artificial neural networks**

Xiaonan Liu^1^, Yong Nie^1#^, and Xiao-Lei Wu^1, 2, 3#^

^1^ College of Engineering, Peking University, Beijing 100871, China

^2^ Institute of Ocean Research, Peking University, Beijing 100871, China

^3^ Institute of Ecology, Peking University, Beijing 100871, China

^#^Corresponding author: Research Scientist, College of Engineering, Peking University. Tel: +86 10-62759047; Fax: +86 10-62759047; E-mail: nieyong@pku.edu.cn

^#^Corresponding author: Professor, College of Engineering, Peking University.

Tel: +86 10-62759047; Fax: +86 10-62759047; E-mail: xiaolei_wu@pku.edu.cn

**This additional information contains:**

**Supplementary analysis:** Grouping and Comparison of ASVs_>10%_

**Grouping and Comparison of ASVs_>10%_**

For a more detailed analysis of the impact of relative abundance and occurrence frequency on the predictability of ASVs, we also grouped all ASVs by relative abundance(1) and occurrence frequency(2), and compared the predictability of different groups.

First, grouped by relative abundance, there were 321 ASVs with an average relative abundance above 0.05%(named high-abundance taxa), 172 ASVs with an average relative abundance below 0.005%(named low-abundance taxa), and 1000 ASVs with an average relative abundance between 0.05% and 0.005% (named medium-abundance taxa) in the ASVs_>10%_ sub-community (Table S2). By comparing the predictability of high, medium, and low-abundance taxa, we found that test R^2^_1:1_ of medium-abundance taxa was significantly higher than that of low-abundance taxa and lower than that of high-abundance taxa (Figure S6a). This result suggested that the higher the relative abundance of microbial taxa, the more predictable it is.

Then, we divided the ASVs_>10%_ sub-community into high (appears in more than 50% of samples), medium (appears in 20% to 50% of samples), and low (appears in less than 20% of samples) frequency groups (Table S2). By comparing the predictability of the different groups described above, we found that the test R^2^_1:1_ of the low-frequency group was significantly lower than that of the medium-frequency group, even though the predictive accuracy R^2^_1:1_ of the medium-frequency group and the high-frequency group had no significant differences (Figure S6b). This result shows that the increase in occurrence frequency may also lead to higher predictability of the taxa.

**References**

1. Liu L, Yang J, Yu Z, Wilkinson DM. The biogeography of abundant and rare bacterioplankton in the lakes and reservoirs of China. ISME J. 2015;9(9):2068-77.

2. Dueholm MKD, Nierychlo M, Andersen KS, Rudkjobing V, Knutsson S, Mi DASGC, et al. MiDAS 4: A global catalogue of full-length 16S rRNA gene sequences and taxonomy for studies of bacterial communities in wastewater treatment plants. Nat Commun. 2022;13(1):1908.
