# Supplementary material for: Predicting microbial community compositions in wastewater treatment plants using artificial neural networks
Source: Microbiome. 2023 Apr 28;11:93. doi: 10.1186/s40168-023-01519-9 (PMC10142226; doi:10.1186/s40168-023-01519-9)
Supplement: Supplementary file 3 — Additional file 2: Figure S1. Ranking of importance weights ofenvironmental factors in different alpha-diversities predictive models. FigureS2. a. Comparison ofintra- and inter-group Bray-Curtis similarity between predicted and observedcommunities. b. Average prediction accuracy R21:1of microbial taxa at different taxonomic levels. Figure S3. Environmental factor importance weights andPearson’s correlation coefficients. FigureS4. Correlation ofcorrelation coefficients of environment factors with ASVs>10%subcommunity, skewness, and kurtosis of normalized environment variables withtheir Garson’s connection weights. Figure S5. a. Comparison of predictiveaccuracy R21:1 between low,medium, and high abundance taxa. b. Comparison of predictiveaccuracy R21:1 between low,medium, and high-frequency taxa. c. Correlation of relative abundance with the occurrencefrequency of ASVs. d. Correlation of the R21:1in test sets with the coefficient of variation of ASVs. Figure S6. Comparison ofaverage relative abundance and occurrencefrequency between above, neutral, and below partitions. Figure S7. Fit of theneutral community model (NCM) of above, neutral, and below partitions. Figure S8.The taxonomic composition, average relative abundance, occurrence frequency,and estimated migration rate of core and non-core taxa. Figure S9. Predictionof functional groups with 10 high-weight environmental factors. Figure S10. Fitof the neutral community model (NCM) of high abundance, medium abundance, andlow abundance subcommunities. Figure S11. Changes of mean square errors (MSE) andcoefficients of determination (R2) on the validation set with epochswhen training the model. [file 40168_2023_1519_MOESM2_ESM.docx]

Additional file 2 of the article:
**Predicting microbial community compositions in wastewater treatment plants using artificial neural networks**

Xiaonan Liu^1^, Yong Nie^1#^, and Xiao-Lei Wu^1, 2, 3#^

^1^ College of Engineering, Peking University, Beijing 100871, China

^2^ Institute of Ocean Research, Peking University, Beijing 100871, China

^3^ Institute of Ecology, Peking University, Beijing 100871, China

^#^Corresponding author: Research Scientist, College of Engineering, Peking University. Tel: +86 10-62759047; Fax: +86 10-62759047; E-mail: nieyong@pku.edu.cn

^#^Corresponding author: Professor, College of Engineering, Peking University.

Tel: +86 10-62759047; Fax: +86 10-62759047; E-mail: xiaolei_wu@pku.edu.cn

**This additional information contains:**

**Supplementary Figures:** Fig. S1 to Fig. S11
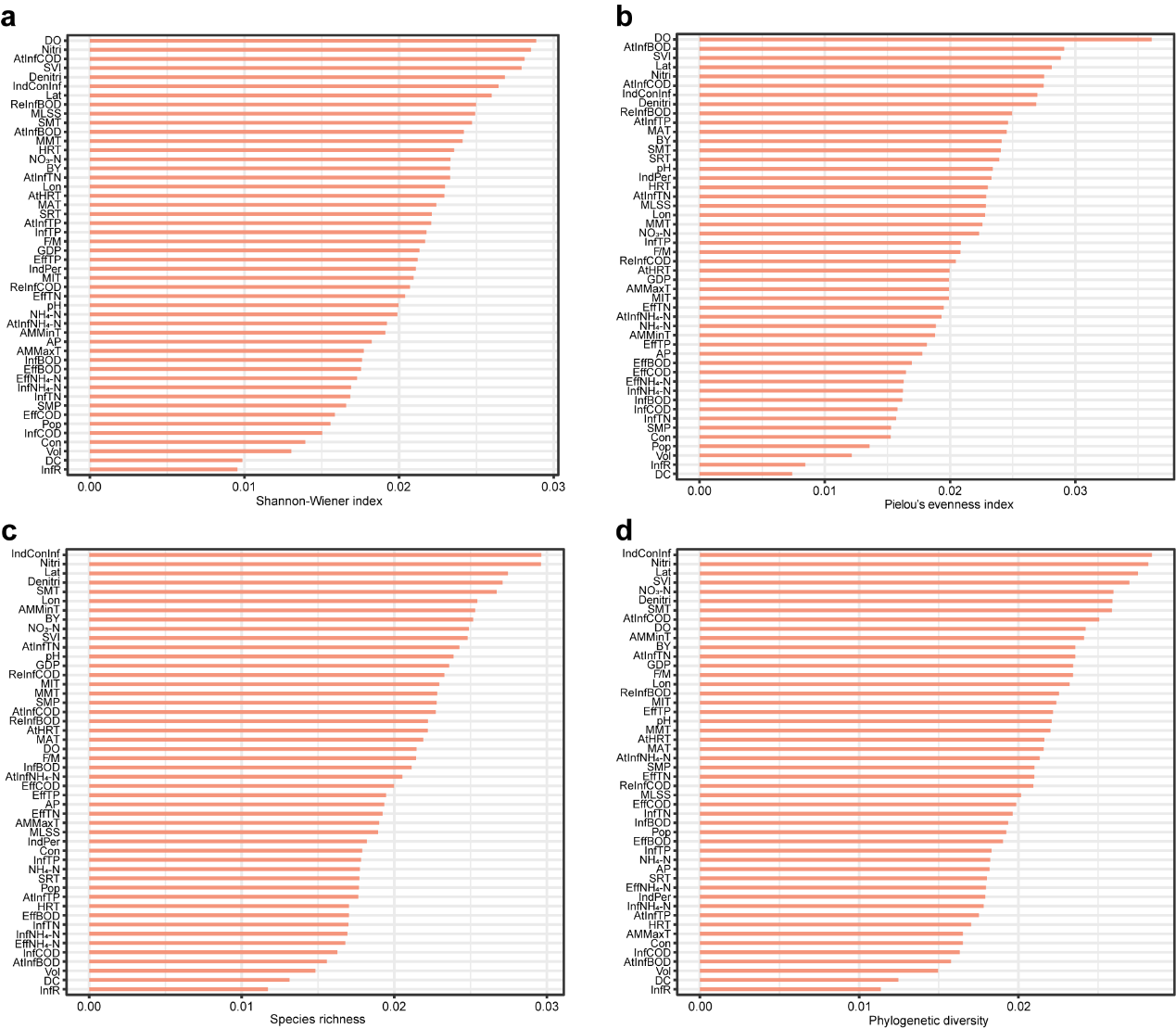


**Figure S1** Ranking of importance weights of environmental factors in different alpha-diversities predictive models.


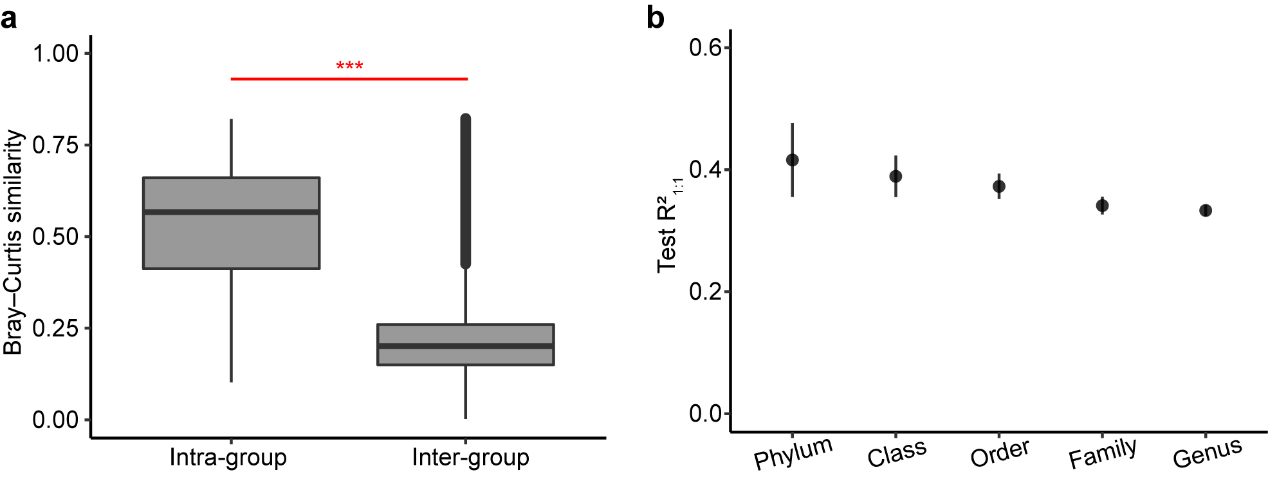


**Figure** **S2** **a.** Comparison of intra-group and inter-group Bray-Curtis similarity between predicted and observed communities. Statistical analysis was performed using a two-sample Student's t-test: ***, p < 0.001. **b.** Average prediction accuracy R^2^_1:1_ of microbial taxa at different taxonomic levels.
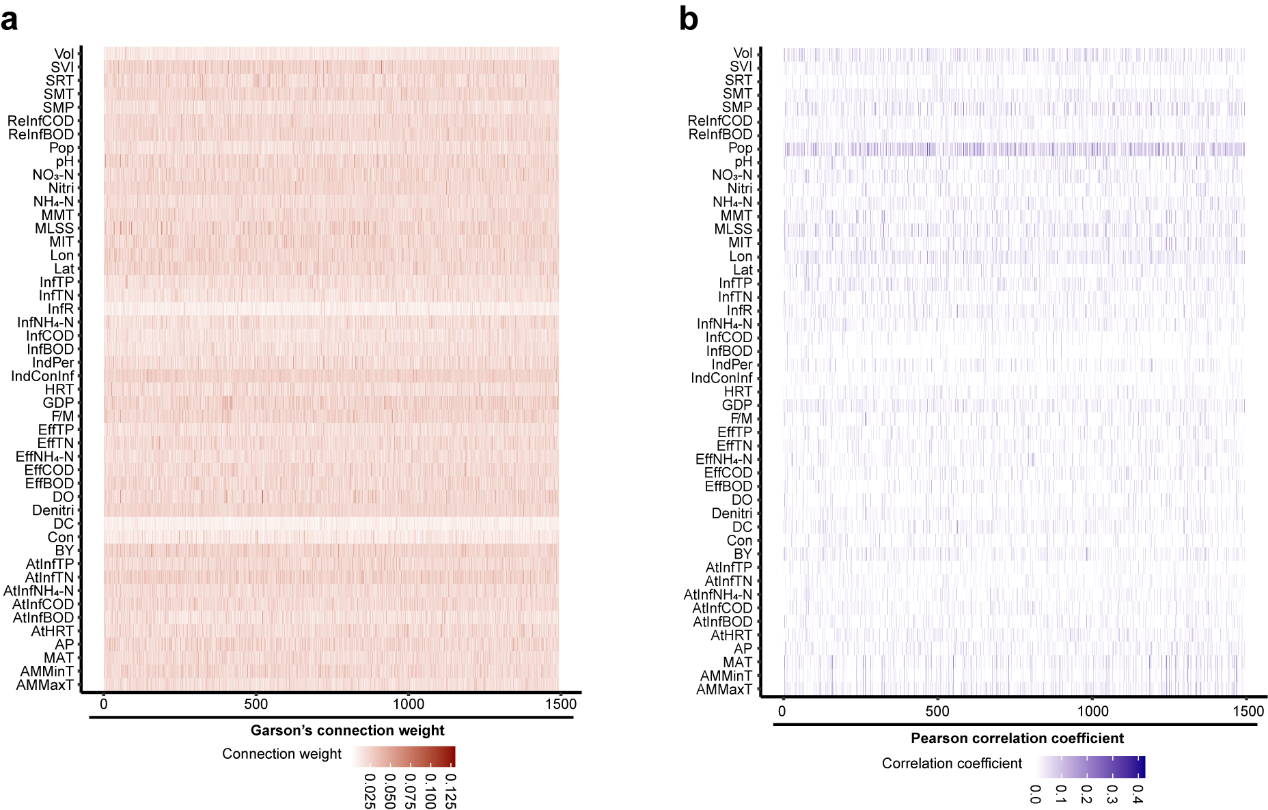


**Figure S3** **a.** Environmental factor importance weights for predicting ASVs_>10%_ sub-community. **b.** Pearson’s correlation coefficients between environmental factors and ASVs_>10%_ subcommunity.
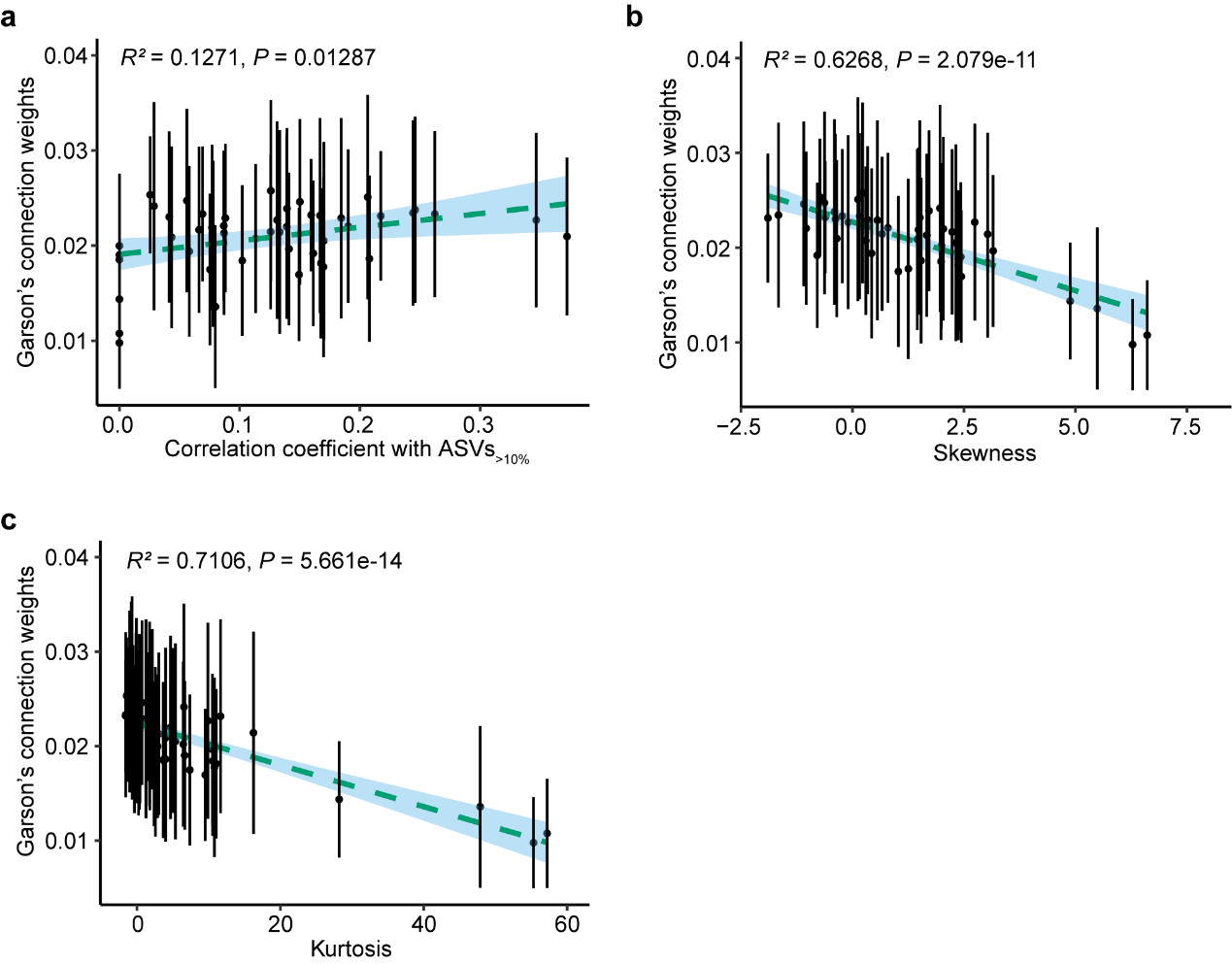


**Figure S4 a.** Correlation of correlation coefficients of environment factors with ASVs_>10%_ subcommunity with their Garson’s connection weights. Correlation of skewness (**b**) and kurtosis (**c**) of normalized environment variables with their Garson’s connection weights. The best fit is shown in the ocean dashed line. The shaded sky region represents the 95% confidence interval for the best-fit line. Points represent average Garson’s connection weights, and error bars show 95% credible intervals of average Garson’s connection weights.


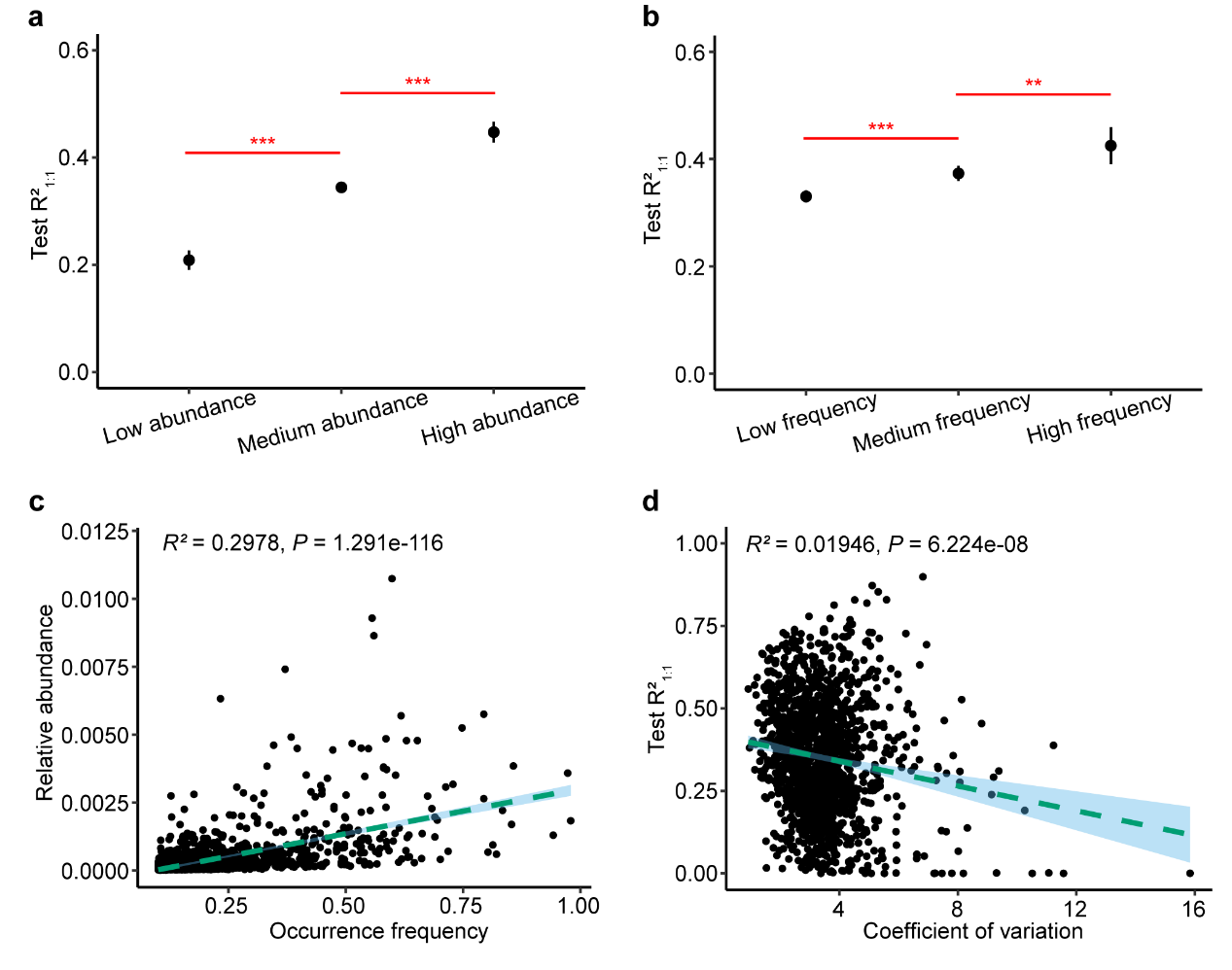


**Figure S5 a.** Comparison of predictive accuracy R^2^_1:1_ between low, medium, and high abundance taxa. **b.** Comparison of predictive accuracy R^2^_1:1_ between low, medium, and high-frequency taxa. **c.** Correlation of relative abundance with the occurrence frequency of ASVs. **d.** Correlation of the R^2^_1:1_ in test sets with the coefficient of variation of ASVs. The date was provided by all ASVs in ASVs_>10%_ sub-community. We reported the R^2^ and P value of the best-fit line in these figures.


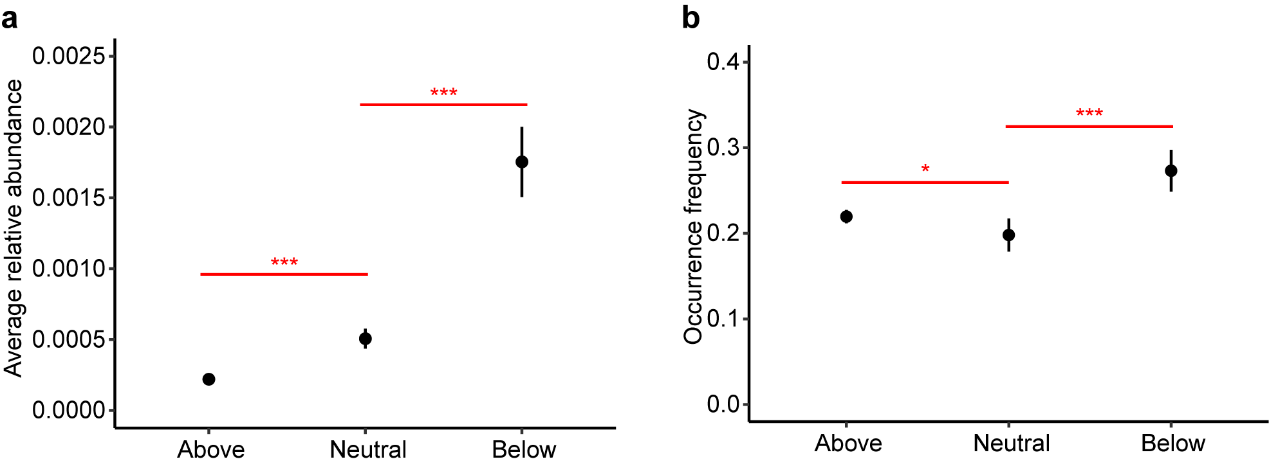


**Figure S6** Comparison of average relative abundance (**a**) and occurrence frequency (**b**) between above, neutral, and below partitions.


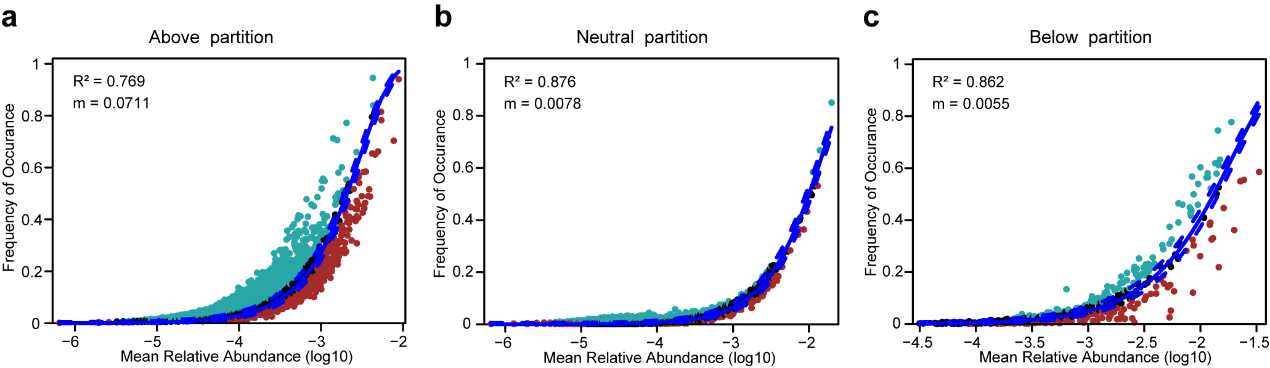


**Figure S7** Fit of the neutral community model (NCM) of above (**a**), neutral (**b**), and below (**c**) partitions.


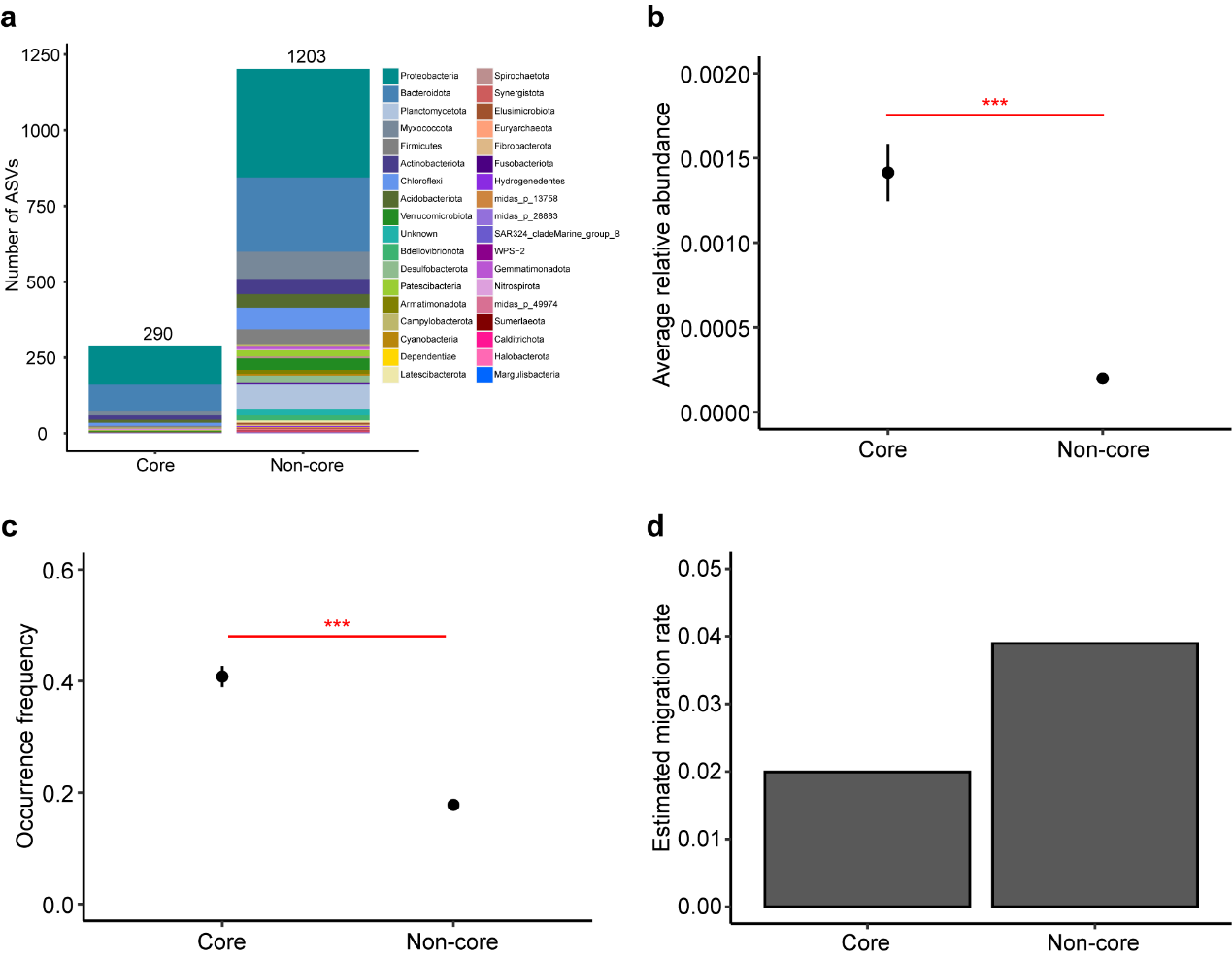


**Figure S8 a.** The taxonomic composition of the core and non-core taxa at the phylum level. Comparison of average relative abundance (**b**) and occurrence frequency (**c**) between core and non-core taxa. **d.** The estimated migration rate of core and non-core taxa.
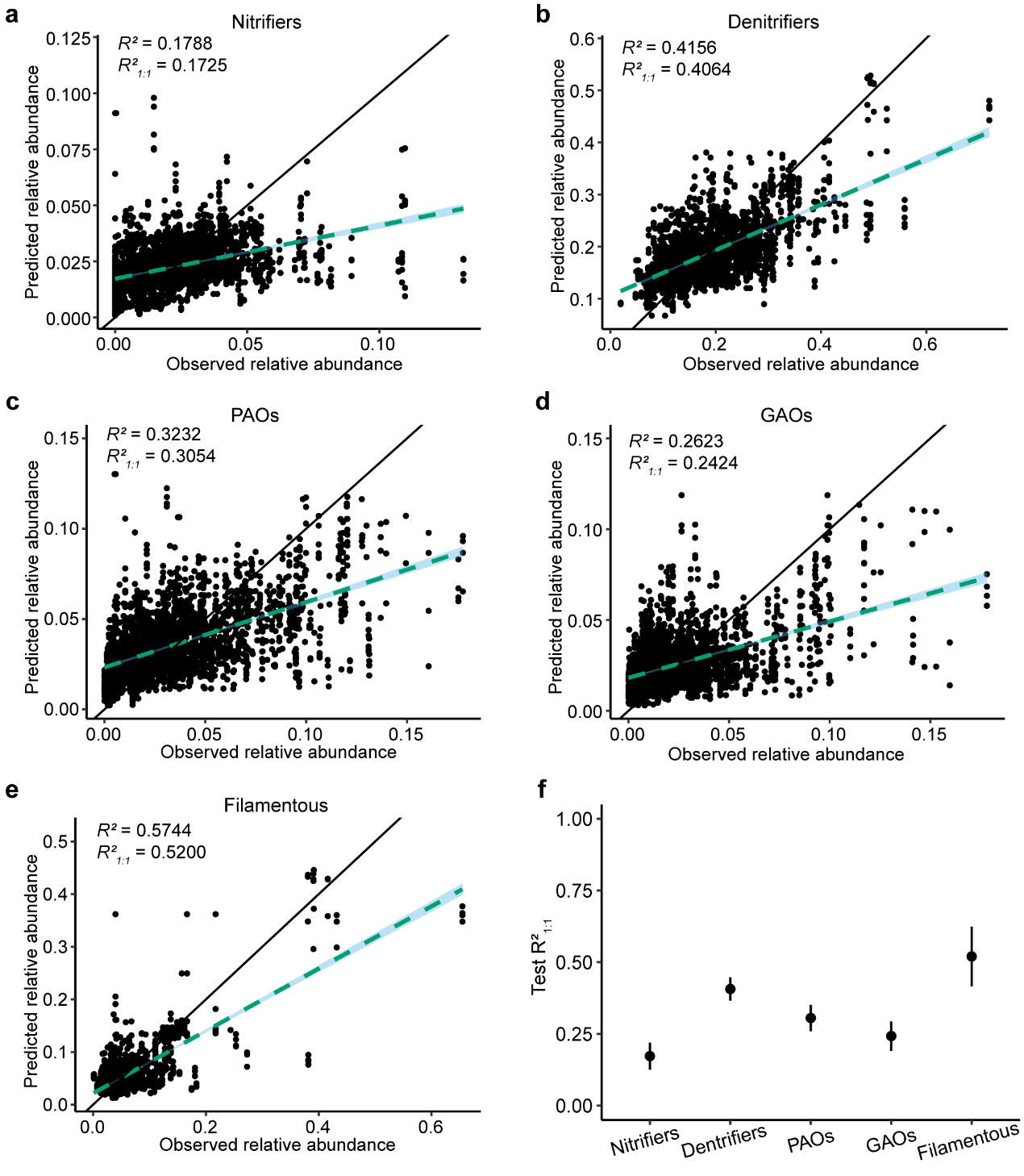


**Figure** **S9** Prediction of functional groups with 10 high-weight environmental factors. Correlations between observed and predicted values of nitrifiers (**a**), denitrifiers (**b**), PAOs (**c**), GAOs (**d**), and Filamentous organisms (**e**). **f.** The test R^2^_1:1_ of nitrifiers, denitrifiers, PAOs, GAOs, and filamentous organisms.


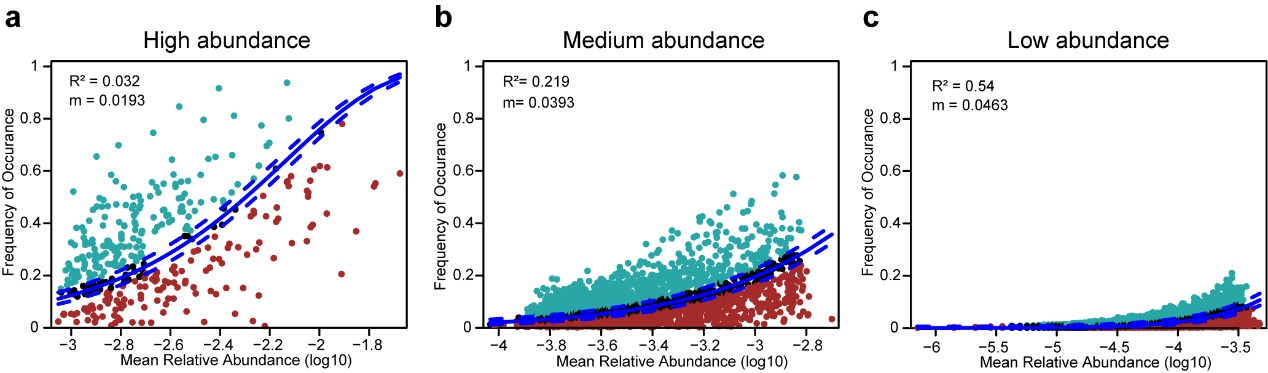


**Figure** **S10** Fit of the neutral community model (NCM) of high abundance (**a**), medium abundance (**b**), and low abundance (**c**) subcommunities.


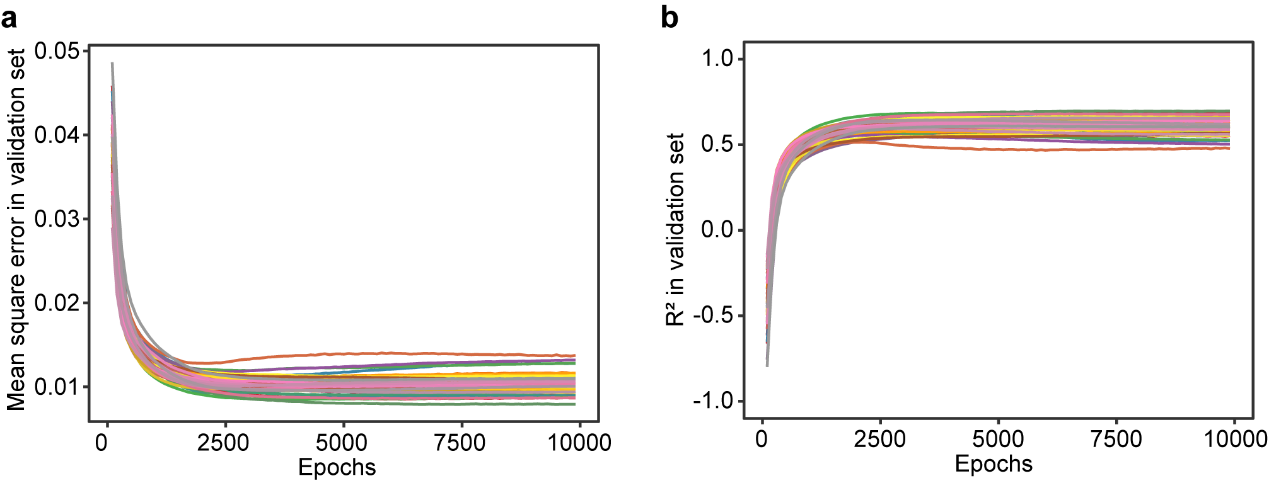


**Figure S11** Changes of mean square errors (MSE) and coefficients of determination (R^2^) on the validation set with epochs when training the model. Take the prediction of the Shanon-Wiener index as an example.
